# Social Support and Linkage to HIV Care Following Routine HIV Testing in a Ugandan Refugee Settlement

**DOI:** 10.1007/s10461-022-03608-6

**Published:** 2022-02-17

**Authors:** Canada Parrish, Erica Nelson, Zikama Faustin, Joshua Stern, Julius Kasozi, Robin Klabbers, Simon Masereka, Alexander C. Tsai, Ingrid V. Bassett, Kelli N. O’Laughlin

**Affiliations:** 1 Department of Emergency Medicine, University of Washington, Seattle, WA, USA; 2 Department of Emergency Medicine, Brigham and Women’s Hospital, Harvard Medical School, Boston, MA, USA; 3 Bugema University, Kampala, Uganda; 4 Department of Global Health, University of Washington, Seattle, USA; 5 United Nations High Commissioner for Refugees, Kampala, Uganda; 6 Medical Teams International, Mbarara, Uganda; 7 Center for Global Health, Massachusetts General Hospital, Boston, MA, USA; 8 Mbarara University of Science and Technology, Mbarara, Uganda; 9 Harvard Center for Population and Development Studies, Cambridge, MA, USA; 10 Department of Medicine, Massachusetts General Hospital, Boston, MA, USA

**Keywords:** HIV, Refugee, Uganda, Linkage to care, Social support, Spatial analysis

## Abstract

We aimed to identify factors associated with linkage to care for individuals newly diagnosed with HIV in a refugee settlement. This study was conducted from October 2018 through January 2020 in Nakivale Refugee Settlement in Uganda. We conducted a cross-sectional survey among individuals accessing routine HIV testing services. The survey included questions on demographic factors, physical and mental health conditions, social support, and HIV-related stigma. We collected GPS coordinates of the homes of individuals newly diagnosed with HIV. Associations with linkage to care were assessed using bivariate and multivariable analyses. Linkage to care was defined as clinic attendance within 90 days of a positive HIV test, not including the day of testing. Network analysis was used to estimate the travel distance between participants’ homes and HIV clinic and to spatially characterize participants living with HIV and their levels of social support. Of 219 participants diagnosed with HIV (out of 5,568 participants screened), 74.4% linked to HIV care. Those who reported higher social support had higher odds of linking to care compared with those who reported lower social support. On spatial analysis, lower levels of social support were most prevalent in Nakivale Refugee Settlement itself, with more robust social support southeast and west of the study area. Social support is a salient correlate of linkage to care for individuals living in refugee settlements and could be the focus of an intervention for improving uptake of HIV care services.

## Introduction

Sub-Saharan Africa is home to 25.7 million people living with HIV [1], and over 18.4 million refugees and internally displaced people [2, 3]. Refugees suffer hardships including difficulty accessing basic needs, disrupted social networks, and increased susceptibility to mental health problems [4-7]. These adversities increase refugee vulnerability to HIV [4, 6, 8], and form unique barriers to accessing medical services and engaging in HIV care [5, 8, 9]. In recent years, HIV testing has successfully expanded in many refugee settlements; linkage to HIV care is the critical next step in reaching the UNAIDS 95-95-95 targets [10] in these communities.

Specific factors which facilitate or impede linkage to HIV care for people residing in refugee settlements are not well known [11]. A previous analysis in a refugee settlement in Uganda demonstrated that only 54% of newly diagnosed adults linked to HIV care within 90 days of diagnosis [12], a significantly lower percentage than non-refugee populations in Africa [13]. Understanding which factors correlate with linkage to HIV care for people with HIV in these settings could help tailor interventions to the unique needs of this population.

Our goal was to evaluate the individual, physical, and social determinants associated with linkage to care for people newly diagnosed with HIV in Nakivale Refugee Settlement in southwestern Uganda. We hypothesized that low social support, mental disorders, and greater distance to the nearest clinic would correlate with failure to link to HIV care.

## Methods

### Study Setting

This study was conducted in Nakivale Refugee Settlement in Uganda. This settlement hosts over 130,000 refugees and asylum seekers from 15 countries, predominantly the Democratic Republic of the Congo (DRC) (51%), Burundi (29%), Somalia (10%) and Rwanda (7%) [14]. Ugandan nationals live in and around Nakivale and access medical services free of charge. A routine HIV testing study at Nakivale Health Center found an HIV prevalence of 4.5% [15]. Free HIV services, including HIV testing and anti-retroviral therapy (ART), are available for refugees and Ugandan nationals within the settlement.

### Study Design

From October 2018 to January 2020, adults were recruited from the outpatient department waiting area at three of the four health centers in Nakivale. To obtain data on potential correlates of HIV care linkage, we collected surveys from willing clients testing for HIV prior to receipt of their HIV test results. The survey was administered in 4 different languages (Runyankore, Kinyarwanda, Kiswahili, English) and entered into mobile REDCap using electronic tablets. Study data were managed using REDCap electronic data capture tools [16] hosted at the Research Information Science & Computing. REDCap (Research Electronic Data Capture) is a secure, web-based application designed to support data capture for research studies, providing: (1) an intuitive interface for validated data entry; (2) audit trails for tracking data manipulation and export procedures; (3) automated export procedures for seamless data downloads to common statistical packages; and (4) procedures for importing data from external sources. The survey was approximately 30 min in length and included questions on demographic factors, health status, mental health conditions, social support and HIV-related stigma. For willing individuals newly diagnosed with HIV, study personnel collected Global Positioning System (GPS) coordinates of their homes.

### Variables of Interest

The outcome of interest was HIV care linkage defined as an HIV clinic visit within 90 days of diagnosis, excluding the day of the initial HIV test. To assess if linkage occurred, data elements were obtained twice monthly from written HIV clinic registers, a method used previously in HIV care cascade research in Nakivale [12].

For self-reported health status, participants were asked, “In general, would you say your health is excellent, very good, good, fair or poor?” Participants were screened for three mental disorders, namely posttraumatic stress disorder (PTSD), anxiety and depression and, as well as for social support and HIV-related stigma. PTSD was assessed using the abbreviated PTSD Checklist; a total score ≥ 14 was considered indicative of PTSD [17]. The depression module of the Patient Health Questionnaire (PHQ-9) was used to screen for depression [18]; a score of ≥ 10 was considered a positive screen for depression [19]. Participants were screened for anxiety using the GAD-7, a 7-item general anxiety disorder scale [20]; a score of ≥ 10 was considered a positive screen for anxiety [21]. These mental health screenings tools have been validated in low-income countries [22], and for use with Ugandan and/or refugee populations [23, 24]. Social support was defined as a binary variable, with *higher* social support defined by scores > 11 on a Brief Social Support Scale (BS-6) instrument that assesses emotional and practical dimensions of social support [25]; scores ≤ 11 indicated *lower* social support. The BS-6 features items, each scored on a four-point Likert-type scale, that include questions such as, “*If you needed it, how often is someone available to help with daily chores if you were sick?*”. HIV-related stigma was assessed using relevant questions from the Uganda Demographic and Health Survey. [26].

Distance from home to clinic was determined for the subset of individuals newly diagnosed with HIV who consented to geocoding. Road networks from Humanitarian Open Street Maps [27] were used to determine potential travel routes. Network analysis was conducted in ArcPro to estimate the travel distance between the participants’ homes and the location of the clinic where they linked to care. To account for homes not on or directly adjacent to the road system, the final travel distance aggregated results from the network analysis and an analysis that calculated the distance between the participants’ homes and the nearest connection point with the road system.

### Data Analysis

Chi-square tests, or Fisher exact tests for categories with small frequencies, were used to estimate associations between categorical variables, such as sociodemographic characteristics like relationship status and education, and secondary outcomes like HIV status and the primary outcome of linkage to HIV care. T-tests were used for continuous variables such as age and distance to clinic, comparing HIV positive and negative individuals and those who linked and those who did not link to care. We also used logistic regression to calculate unadjusted and adjusted odds ratios (ORs) and 95% confidence intervals to assess the association between the individual characteristics of interest that were statistically significant in the bivariate analyses and linkage to HIV care.

To anonymize the data and still be able to visualize the distribution of participants with HIV, addresses were geomasked using a random skew algorithm within a maximum/minimum distance buffer that was informed by the population density at each value. A spatial characterization of individuals who tested HIV positive and screened positive for a lower social support, or lack of social support, was undertaken in ArcMap. Kernel density mapping was conducted using the planar method to visualize levels of social support across the study region represented by densities of ‘lack of social support’ per square kilometer.

## Results

Of 5568 participants enrolled in the study, most were refugees or asylum seekers (65%) with the predominant countries of origin being the Democratic Republic of Congo (24%), Rwanda (23%), and Burundi (17%). Approximately one-third (36%) of the participants were Ugandan nationals. Among all participants, 219 tested positive for HIV (3.9% of all participants and 2.2% of refugees tested). Several statistically significant differences between those who tested positive and negative for HIV were identified (Table 1). Notably, those who tested positive were more likely to be female, more likely to be a Ugandan national, more likely to be divorced/separated/widowed, and less likely to report being in good health. The HIV positive group had a higher proportion of individuals who screened positive for PTSD (50.7% vs. 43.2%, χ^2^ = 4.73; *p* = 0.03).

**Fig. 1:**
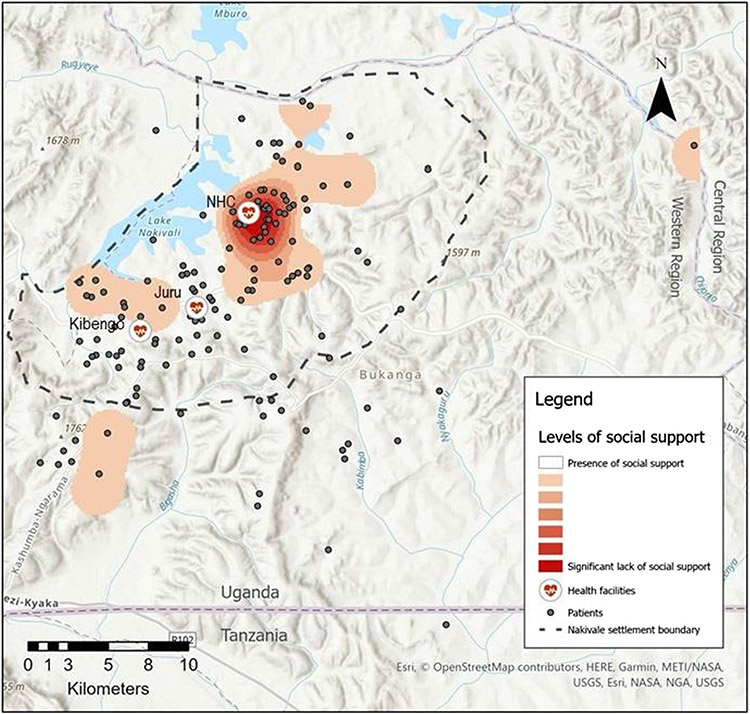
Kernel density map of levels of social support for participants who tested positive for HIV juxtaposed with all participants who tested positive for HIV, health care facilities (including Nakivale Health Center [NHC], Juru Clinic, and Kibengo Clinic), and the Nakivale settlement boundary

Among the participants newly diagnosed with HIV, 74.4% linked to care (defined as attend the HIV clinic within 90 days of the day of diagnosis excluding the day of diagnosis). The distance traveled to clinic varied by the health center where participants initially tested for HIV. This distance was not associated with linkage to care in bivariate analyses. For the 145 HIV positive participants for whom we were able to collect GPS data, the median distances traveled ranged from 3.4 km (2.1 miles) to 10.0 km (6.2 miles); with maximum distances up to 54.8 km (34.2 miles) (Fig. 1). Juru Health Center had a relatively small catchment area with a maximum distance traveled to clinic of 13.2 km (8.2 miles). Conversely, Nakivale and Kibengo HIV clinics demonstrated large catchment areas with some individuals crossing regional and national borders to obtain care. For participants for whom we had home addresses and linked to care, 23.8% did not link to care at the clinic nearest their home.

Of the individual factors that were assessed including demographic characteristics, physical and mental health, social support and HIV-related stigma, only social support was associated with linkage to care (Table 1). A larger proportion of participants who linked to care reported higher social support compared with those who did not link to care (89.4% vs. 78.2%, *p* = 0.03). We found that those who reported higher social support had more than two times the odds of linking to care compared with those who reported lower social support (OR = 2.36, 95% CI: 1.05–5.34). This association remained statistically significant when adjusting for individual characteristics such as sex, age, refugee status, relationship status, and level of education.

Given our findings regarding social support and its association with linkage to care, we conducted a secondary analysis to explore the determinants of social support among HIV positive individuals. In a multivariable logistic regression model restricted to HIV positive individuals (*N* = 219) including individual demographic characteristics of sex, age, refugee status, relationship status, and education level, we found that education was a statistically significant, independent predictor of social support. Those who had some formal education versus no formal schooling had significantly increased odds of reporting higher social support compared to those with no formal schooling (OR = 4.09, 95%CI: 1.52–10.98 and OR = 6.14; 95%CI 1.12–33.71 for some primary and beyond primary school, respectively).

The spatial characterization of social support among HIV positive individuals can be seen juxtaposed with the geomasked locations of residence, health care facilities, and the Nakivale settlement boundary in Fig. 1. This kernel density analysis demonstrates significantly low levels of social support in the Nakivale Refugee Settlement itself, and more robust social support among HIV positive participants in the south-east of the study region and in a large swath of the west.

## Discussion

Almost three-quarters of those testing positive for HIV were linked to care within 90 days of diagnosis. We found that most of the demographic, physical and mental health factors of interest were not associated with linkage to care, with the exception of social support. Those with greater social support were more likely to link to care compared to those with lower social support. In our analyses exploring factors related to social support, we found that more years of schooling was significantly associated with increased social support. It is important to note that although the prevalence of mental health issues was relatively high in our study (approximately 43% of all participants screened positive for PTSD, 25% for depression and 23% for anxiety), these estimates are comparable or lower than that observed in other studies in similar and nearby regions. [28-31].

In the spatial analyses, we found that participants did not consistently link to HIV care at the closest health center to their primary residence and the distance traveled to clinic was highly variable both within and across sites. We did not collect data about why a participant may not have linked to care at their nearest clinic. However, prior work in this region suggests some people decline to attend the clinic nearest to their home to decrease the possibility of involuntary HIV status disclosure to people in their community [32, 33]. The Kernel density mapping illustrated that social support amongst HIV positive participants was lowest in Nakivale settlement and highest in the south of the study region. These findings may be reflective of differing proportions of Ugandan nationals in the study areas. Ugandan nationals likely had less disrupted social networks as they did not flee their country of origin like refugees, and may have more social support given longstanding social ties. Alternatively, the southern area of the study region is more rural, whereas Nakivale has the greatest population density. It may be that those living in more rural settings have stronger ties to their family and neighbors and benefit from that social support.

This study represents novel work in evaluating linkage to care barriers and facilitators for individuals residing in and around refugee settlements. Our finding regarding social support as a salient determinant of linkage to care suggests that interventions to bolster social support may be beneficial in increasing linkage to care and improving HIV outcomes more generally [34]. This is echoed in previous qualitative work in Nakivale, demonstrating that “*Emotional support, particularly acceptance and encouragement, helped refugees overcome these fears [of an HIV diagnosis] to proceed with treatment*. [35].

Interpretation of our findings is subject to certain limitations. First, the sample was non-random and not necessarily representative of the entire settlement community. For example, pregnant women more commonly access HIV testing from the maternity ward and therefore would have been less likely to enroll in this study. Second, the study population includes refugees and Ugandan nationals accessing HIV testing services in the refugee settlement. As such, the findings may not be generalizable to more homogenous refugee populations in Sub-Saharan Africa. Additionally, it is possible that some study participants that we classified as not linking to care could have linked to care at a clinic outside of the Nakivale catchment area, which would have biased our estimated linkage rates. Finally, only a subset (66%) of the participants had GPS data available for the spatial analyses.

This research suggests that social support appears to increase linkage to care for individuals living in refugee settlements, and linkage to care in this study was more frequent than in previous studies in this settlement [12], suggesting that linkage has improved over time. However, this rate is below the national Ugandan reports of 88.4% of adults living with a known HIV status who report being in treatment [36], indicating that there is room for improvement in these refugee settlement communities. Linking HIV positive individuals to care can be challenging, and rates of care linkage are highly variable across regions in sub-Saharan Africa. The likelihood of timely linkage is often dependent on the modality of testing (facility- vs. community-based) with linkage estimates ranging from 26% to 95%. [37] Identifying factors that promote or inhibit linkage to care is critical for the attainment of the UNAIDS fast track goal of having 95% of those diagnosed with HIV to be engaged in care [10]. Interventions which strengthen existing support systems or establish new networks of support for individuals living with HIV may have the potential to improve progression along the HIV care continuum in refugee settlements and bolster national HIV programming to improve linkage in testing programs with sub-optimal linkage rates. [37].

Improving linkage to HIV care is the next important step after the expansion of HIV testing programs. This study demonstrated that social support is associated with timely linkage to care. Leveraging social support networks and resources may improve linkage to care for those newly diagnosed with HIV in refugee settlements in Uganda.

## Figures and Tables

**Table 1:** Comparison of HIV testing participants (among all participants) and those who did and did not link to care (among those living with HIV)

Individual characteristics	Tested HIV + *N* = 219	Tested HIV- *N* = 5,349	Test statistic*	*p*-value	Linked to care *N* = 163	Not linked to care *N* = 56	Test statistic***	*p*-value
*Demographics*								
Female, N (%)	144 (66.1)	2887 (54.0)	12.25	<0.01	108 (66.3)	36 (65.5)		0.91
Age in years, mean (SD)	33.0 (10.4)	32.2 (11.4)	1.10	0.27	33.2 (10.5)	32.5 (10.5)	−0.40	0.69
Refugee Status, *N* (%)			92.57	<0.01				0.36
Refugee	79 (36.2)	3503 (65.8)			59 (36.2)	20 (36.4)		
Ugandan National	132 (60.6)	1752 (32.9)			99 (60.7)	33 (60.0)		
Asylum seeker	6 (2.8)	28 (0.5)			5 (3.1)	1 (1.8)		
Non-Ugandan national	1 (0.5)	41 (0.8)			0 (0.0)	1 (1.8)		
Relationship Status, *N* (%)			37.12	<0.01				0.52
Married/living together	109 (50.0)	2943 (55.1)			82 (50.3)	27 (49.1)		
Divorced/separated/widowed	86 (39.4)	1231 (23.1)			66 (40.5)	20 (36.4)		
Single	23 (10.6)	1163 (21.8)			15 (9.2)	8 (14.5)		
Education, *N* (%)			15.04	<0.01				0.99
No school	42 (19.3)	1147 (21.5)			31 (19.5)	11 (20.0)		
Some primary	119 (54.6)	2702 (50.6)			89 (54.1)	30 (54.5)		
Completed primary	30 (13.8)	435 (8.1)			23 (14.5)	7 (12.7)		
Primary +	27 (12.4)	1061 (19.9)			20 (11.9)	7 (12.7)		
*Health and well-being*								
Self-reported health status, *N* (%)			50.56	<0.01				0.67
Excellent	4 (1.8)	93 (1.7)			3 (1.8)	1 (1.8)		
Very good	24 (11.0)	988 (18.5)			18 (11.0)	6 (10.9)		
Good	71 (32.6)	2526 (47.3)			49 (30.1)	22 (40.0)		
Fair	76 (34.9)	1211 (22.7)			58 (35.6)	18 (32.7)		
Poor	43 (19.7)	517 (9.7)			35 (21.5)	8 (14.5)		
PTSD, *N* (%)	109 (50.7)	2289 (43.2)	4.73	0.03	81 (50.6)	28 (50.9)		0.97
Depression, *N* (%)	55 (25.5)	1288 (24.8)	0.05	0.82	44 (27.3)	11 (20.0)		0.28
Anxiety, *N* (%)	49 (22.9)	1197 (22.9)	0.01	0.99	41 (25.6)	8 (14.8)		0.10
High social support *N* (%)	187 (86.6)	4,611 (88.2)	0.54	0.46	144 (89.4)	44 (78.2)		0.03
HIV-related stigma, *N* (%)			3.65	0.16				0.51
None/little	99 (49.0)	2217 (42.6)			77 (51.0)	22 (43.1)		
Moderate	95 (47.0)	2677 (53.9)			69 (45.7)	23 (51.0)		
Extreme	8 (4.0)	176 (3.5)			5 (3.3)	3 (5.9)		
*Testing location*								
Health center			7.40	0.03				0.85
Nakivale	119 (54.6)	2675 (50.0)			90 (55.2)	29 (52.7)		
Juru	38 (17.4)	1366 (25.5)			29 (17.8)	9 (16.4)		
Kibengo	61 (28.0)	1307 (24.4)			44 (27.0)	17 (30.9)		
Distance to clinic (km), mean (SD)	N/A	N/A		N/A	8.0 (8.6)	6.7 (5.9)	−0.72	0.47

* Chi-square test for categorical variables; two-sample t test for continuous variables

*** Fsher’s exact test for categorical variables (test statistic not applicable); two-sample t test for continuous variables

## Data Availability

Not available due to stipulations in the ethics approval process.
